# Role of Serum Magnesium on Insulin Resistance in Overweight and Obese Children

**DOI:** 10.7759/cureus.69622

**Published:** 2024-09-17

**Authors:** Sowmya Gadiparthi, Siddarameshwar Kalyanshettar, Mallanagouda Patil, Shankargouda V Patil

**Affiliations:** 1 Pediatrics, Shri B. M. Patil Medical College Hospital and Research Centre, BLDE (Deemed to be University), Vijayapura, IND

**Keywords:** childhood obesity, homa, insulin, magnesium, overweight

## Abstract

Background

Childhood obesity has become a global epidemic, with projections indicating it could affect 25% of children under 16 by 2050. This rise poses significant health risks, including early onset of type 2 diabetes and cardiovascular diseases. Recent research has shown a correlation between insulin action and magnesium levels, highlighting the link between low serum magnesium levels and obesity. This study aimed to investigate the relationship between serum magnesium levels and obesity in children aged 5-16 years, to compare overweight and obese adolescents with age- and gender-matched controls, and to determine the association between insulin resistance and decreased serum magnesium levels.

Methodology

At a tertiary hospital, this 1.5-year cross-sectional comparative study investigated the relationship between magnesium levels in the blood and insulin resistance in children aged 5-16. Cases met the 2015 revised Indian Academy of Pediatrics (IAP) growth charts for overweight or obesity in comparison to age-matched healthy controls. Exclusions included secondary causes of obesity and medical complications affecting magnesium. Assessments involved clinical history, examination, and fasting blood tests for insulin resistance indices.

Results

Compared to the controls, overweight children showed that mean serum magnesium levels are lower (1.03 mEq/l vs. 1.85 mEq/l, p<0.001); they were also reflected in higher anthropometric measures, which include body mass index (BMI) and body fat percentage. Cases had significantly higher metabolic markers like fasting insulin, Homeostasis Model Assessment of Insulin Resistance (HOMA-IR) index, and lipid profiles, whereas systolic blood pressure although showing recognized differences did not significantly differ.

Conclusion

The study underscored the importance of detecting magnesium levels on insulin resistance in overweight and obese children.

## Introduction

The number of obese kids had grown from 11 million in 1975 to 124 million by 2016 when there was an alarming increase in obesity cases among children globally [1]. This trend is projected to continue, potentially affecting up to 25% of children under 16 by 2050, highlighting it as a significant public health concern [2]. Childhood overweight results in many health problems, among them sleep apnea and gastrointestinal and musculoskeletal issues, in addition to hastening the emergence of type 2 diabetes and cardiovascular diseases. Therefore, urgent interventions, specifically from health systems, are required for the control and reduction of the dangers of non-communicable diseases (NCDs) related to obesity [3].

Magnesium (Mg^2+^), the body's fourth most abundant cation, ranging between 1.7 mEq/L and 2.5 mEq/L, is crucial in numerous enzymatic functions essential for glucose and energy metabolism. Roughly 60% of magnesium is stored in bones, 40% in soft tissues, and less than 1% in the bloodstream. Magnesium is a crucial cofactor for many enzymes involved in metabolic pathways, neurotransmitter release, brain function, and muscle contraction and relaxation [4,5]. Magnesium acts as a second messenger for insulin action on tyrosine kinase receptors. Phosphorylase B kinase activity, which releases glucose 1-phosphate from glycogen, is also affected by magnesium. Glucose translocation into the cell is affected by magnesium via its effect on glucose transporter protein activity 4 (GLUT4) [6].

Research indicates a significant correlation between insulin action and magnesium levels [5,6]. Adults with insulin resistance often exhibit reduced insulin production, poor glucose tolerance, and low blood and intracellular magnesium concentrations [7]. Large epidemiologic studies suggest a lower blood and dietary magnesium level is linked to a higher risk of type 2 diabetes [8,9]. There is limited data on the association between low serum magnesium levels and obesity in tier 2 cities of North Karnataka. This study aimed to conduct a comparative analysis of magnesium levels in overweight and normal-weight children, with the primary objective of investigating the potential of magnesium supplementation in mitigating insulin resistance among overweight children.

## Materials and methods

The study was a cross-sectional comparative study conducted at the pediatric OPD of Shri B. M. Patil Medical College Hospital and Research Centre, BLDE (Deemed to be University), in Vijayapura, India, lasting one year, from September 2022 to September 2023. The institutional ethical clearance was obtained from the Ethical Committee of BLDE (Deemed to be University) (approval number: BLDE(DU)/IEC/652/2022-23). The study included children between the ages of five and 16 who satisfied the diagnostic criteria for overweight or obesity according to the 2015 revised Indian Academy of Pediatrics (IAP) growth charts [10] and healthy individuals who participated in immunization clinics and matched age and gender as cases and controls, respectively. Children who have secondary causes of obesity, such as those with diabetes mellitus, hypothyroidism, genetic syndrome, or Cushing syndrome, and medical complications that increase their risk of developing hypomagnesemia, such as chronic kidney and liver disease, were excluded.

Sample size

The sample size calculated using the G*Power 3.1.9.4 software determined that 110 participants (55 per group) are required to achieve 99% power in detecting differences in serum magnesium levels between overweight (mean=2.12, SD=0.33) and normal-weight (mean=2.56, SD=0.24) individuals. This calculation was based on a t-test for independent means, assuming equal group sizes, to ensure robust statistical analysis of the difference between the two groups.

Data collection

Subjects meeting the inclusion criteria were admitted to the pediatric medical ward. Informed consent was obtained, and the following assessments were conducted: an extensive clinical history encompassing the duration of obesity, medication usage, family history of obesity, lipid disorders, and hypertension was taken. The clinical examination included measurements of weight, height, waist circumference, and blood pressure. After overnight fasting for 12 hours, 5 ml of venous blood was drawn, and the following parameters were measured at once. Laboratory investigations involved fasting blood tests to measure blood sugar, glycated hemoglobin (HbA1C), serum insulin, lipid profile, sodium, potassium, calcium, and magnesium levels. When both cases and controls were admitted to the pediatric ward, the above variables, including history taking and laboratory investigations, were sent simultaneously. Body fat percentage was determined by impedance analysis using a Bodystat analyzer. The insulin resistance index was calculated using the Quantitative Insulin Check Index (QUICKI) and Homeostasis Model Assessment of Insulin Resistance (HOMA-IR) as estimates [11]. Overweight and obesity were defined according to the 2015 revised IAP growth charts, with overweight classified as a body mass index (BMI) ranging from the 23rd to 27th percentiles of the adult population. BMI was calculated by dividing weight in kilograms by height in meters squared (kg/m^2^). The insulin resistance indices were computed as follows: HOMA-IR was determined by dividing the fasting glucose concentration (mmol/L) by the fasting insulin concentration (μu/ml), while QUICKI was computed as 1/(logarithm of glucose concentration (mg/dl) plus logarithm of fasting insulin concentration (μu/ml)) [11].

Statistical analysis

The information was entered into Microsoft Excel, and IBM SPSS Statistics for Windows, Version 20.0 (Released 2011; IBM Corp., Armonk, New York, United States), was used to conduct the statistical analysis. Descriptive statistics were calculated to provide means, standard deviations, counts, percentages, and diagrams. For ordinarily distributed continuous variables, the independent sample t-test was utilized; for non-normally distributed variables, the Mann-Whitney U test was applied; and for categorical variables, the chi-squared/Fisher's exact test was applied. Consideration was given to significance at a p-value of 0.05 (two-tailed).

## Results

The mean ages of the cases and controls were 140.51±29.82 and 146.18±31.25, respectively, with a t-value of 0.97406 and a p-value of 0.323 (Table 1). The mean systolic blood pressures of the cases and controls were 98.36±7.64 and 100.05±7.39, respectively, with a p-value of 0.223. The mean diastolic blood pressures of the cases and controls were 65.27±5.04 and 65.82±4.98, respectively, with a p-value of 0.567. The mean waist circumferences of the cases and controls were 86±8.9 and 59±6.8, respectively, with a p-value of <0.001.

**Table 1:** Age of study participants SD: standard deviation

Population	Mean±SD	T-value	P-value
Cases	140±29.82	0.97406	0.323
Controls	146±31.25		

Table 2 shows the demographic characteristics of the study participants.

**Table 2:** Demographic characteristics of the study participants

Variable	Case	Control	Chi-squared statistics	P-value
Gender				
Male	28	28	0	1
Female	27	27		
Family history of				
Obesity	25	29	1.2721	0.52935
Diabetes	19	23		
Hypertension	32	26		

The mean BMI distribution and percentage body fat distribution exhibited significant differences between cases and controls (p<0.001) (Table 3). 

**Table 3:** Mean BMI and percentage of body fat BMI: body mass index; SD: standard deviation

Variable	Case		Control		T-value	P-value
	Mean	SD	Mean	SD		
BMI (kg/m^2^)	31.33	4.35	18.04	2.3	20.0301	<0.001
Percentage of body fat	43.31	4.20	21.46	3.59	29.2558	<0.001

There is no disparity between fasting glucose levels and HbA1c among cases and controls. Fasting insulin showed statistically significant values. The mean HOMA-IR of the cases and controls were 4.35±1.08 and 1.78±0.42, respectively, with a p-value of <0.001. The mean QUICKI of the cases and controls were 0.309±0.0103 and 0.351±0.131, respectively, with a p-value of <0.001 (Table 4).

**Table 4:** Fasting glucose, HbA1c, and fasting insulin levels, HOMA-IR, and QUICKI of the study participants HbA1c: glycated hemoglobin; SD: standard deviation; HOMA-IR: Homeostasis Model Assessment for Insulin Resistance; QUICKI: Quantitative Insulin Check Index

Variable	Case		Control		T-value	P-value
	Mean	SD	Mean	SD		
Fasting glucose (mmol/l)	5.04	0.58	5.03	0.56	0.0267	0.955
HbA1c (%)	5	0.55	4.91	0.59	0.7538	0.453
Fasting insulin (µu/l)	19.53	4.59	7.98	1.74	17.4061	<0.001
HOMA-IR	4.35	1.08	1.78	0.42	16.4582	<0.001
QUICKI	0.309	0.0103	0.351	0.0131	18.7516	<0.001

The mean serum cholesterol, triglycerides, and low-density lipoproteins (LDL) were high and serum high-density lipoprotein (HDL) levels were lower in overweight children compared to controls. Overweight children have a mean serum magnesium level of 1.03 mEq/l, while controls exhibit a notably higher mean of 1.85 mEq/l (p<0.001). There was a non-significant difference in serum calcium (p=0.552) and serum sodium (p=0.359) between cases and controls (Table 5).

**Table 5:** Blood investigations LDL: low-density lipoproteins; HDL: high-density lipoproteins; SD: standard deviation

Variable	Case		Control		T-value	P-value
	Mean	SD	Mean	SD		
Cholesterol (mg/dl)	179.64	11.43	132.16	12.24	21.0162	<0.001
Triglycerides (mg/dl)	152.12	14.38	93.6	17.33	19.2644	<0.001
LDL (mg/dl)	124.89	2.81	85	14.41	20.1402	<0.001
HDL (mg/dl)	24.65	3.08	31.73	8.05	6.0843	<0.001
Serum magnesium (mEq/l)	1.03	0.54	1.85	0.56	7.7751	<0.001
Serum calcium (mEq/l)	10.05	0.57	10.11	0.62	0.4821	0.552
Serum sodium (mEq/l)	140.91	2.81	140.44	2.64	0.8802	0.359

Table 6 shows the correlation between serum magnesium and HOMA-IR and QUICKI.

**Table 6:** Correlation between serum magnesium and HOMA-IR and QUICKI HOMA-IR: Homeostasis Model Assessment for Insulin Resistance; QUICKI: Quantitative Insulin Check Index

Serum magnesium	HOMA-IR		QUICKI		
	Pearson correlation	P-value	Pearson correlation	P-value	
Cases	0.011	0.939	0.030	0.827	
Controls	0.220	0.107	0.192	0.159	

## Discussion

The primary objective of the current investigation was to examine the association between insulin resistance and serum magnesium levels in overweight children aged 5-16. The age distribution analysis revealed a non-significant difference in mean age between cases (140.51 months) and controls (146.18 months), with a p-value of 0.323. This suggests a balanced age distribution between the groups, minimizing the potential influence of age as a confounding factor in subsequent analyses.

The present study demonstrates a balanced gender distribution with 51% males and 49% females in both case and control groups, confirmed by a p-value of 1.000. This even representation contrasts with the study by Huerta et al. [11], which found a higher proportion of females (62.5%) in both groups. While both studies maintain consistency between cases and controls, the present study's larger sample size and more balanced gender ratio may offer greater statistical power and better population representation.

According to the findings, serum magnesium levels differ significantly among cases and controls. Overweight children have a mean serum magnesium level of 1.03 mEq/l, while controls exhibit a notably higher mean of 1.85 mEq/l (p<0.001). This substantial discrepancy raises intriguing implications for the potential association between serum magnesium levels and insulin resistance in overweight children (as shown in Table 6).

Low serum magnesium levels in overweight individuals have been previously linked to insulin resistance, a precursor to type 2 diabetes. Magnesium plays a crucial role in insulin signaling and glucose metabolism, and deficiency may impair insulin sensitivity. The findings in this study align with existing literature, suggesting that lower serum magnesium levels in overweight children may contribute to the observed insulin resistance.

Çelik et al. [12], a study done in 2011 using a sample of 203 individuals, found that serum magnesium levels were significantly lower in insulin-resistant obese individuals compared to controls (p=0.014). Another interesting observation from their study was a strong association between blood magnesium levels and BMI-SDS in the insulin-sensitive obese group, emphasizing a potential link between magnesium status and insulin resistance. Huerta et al. [11] reported substantially lower serum magnesium levels in obese children compared to their lean counterparts (0.748±0.015 mmol/l vs. 0.801±0.012 mmol/l, p=0.009). Additionally, they identified a positive correlation between QUICKI and serum magnesium and an inverse relationship between serum magnesium and fasting insulin. The reduced dietary magnesium consumption in obese children further underscores the importance of magnesium in metabolic health.

In the present study, Jose et al. [13] and Suliburska et al. [14] demonstrate significantly lower serum magnesium levels in overweight/obese individuals than those of normal weight. The present study quantified this difference in children (1.03 mEq/l vs. 1.85 mEq/l, p<0.001), providing specific values not mentioned in the other studies. All three studies consistently show associations between reduced magnesium levels and various metabolic parameters. In terms of insulin resistance, the present study and both comparative studies found correlations with insulin resistance markers. The present study specifically noted higher fasting insulin and HOMA-IR in overweight cases. Regarding anthropometric measures, the present study and Jose et al. observed relationships between magnesium levels and BMI, with the present study also including body fat percentage data. The present study found significantly higher lipid profiles in overweight children, a detail not mentioned in the summaries of the other two studies. Jose et al. [13] noted an inverse relationship in blood pressure, while the present study found only non-significant differences in systolic blood pressure. It's worth noting that Suliburska et al. [14] included serum zinc analysis and focused on adolescents, while the present study provided more comprehensive metabolic data in children. Overall, all three studies support the relationship between low magnesium levels and metabolic disturbances in obesity, with the present study offering the most comprehensive analysis of metabolic parameters in children specifically.

Guerrero-Romero and Rodríguez-Morán [15] found that individuals with metabolic syndrome had significantly lower blood magnesium concentrations compared to healthy controls (1.8±0.3 mg/dl vs. 2.2±0.2 mg/dl, p<0.00001). The study highlighted the independent association between low blood magnesium concentrations and the presence of metabolic syndrome, with hypertension and dyslipidemia being the most strongly associated components. The study by Hassan et al. [16] reinforced the consistent trend, demonstrating considerably lower blood magnesium levels in overweight and obese children compared to normal-weight controls (2.08±0.211 mg/dl vs. 2.55 ± 0.155 mg/dl). This finding potentially connects magnesium deficit to obesity severity because BMI inversely correlates significantly with serum magnesium levels. In overweight children, variations observed in serum magnesium levels reveal the significance of thinking about their magnesium statuses during interventions that aim at controlling insulin resistance within this group.

To establish a more definitive causal link and examine the potential advantages of magnesium supplementation in this regard, additional research is needed, including longitudinal studies or interventional trials. The limitations of this research need to be borne in mind, including this being a cross-sectional study and the need for more studies to understand better the complicated interaction of serum magnesium levels, insulin resistance, and being overweight.

## Conclusions

The results of the study showed a significant relationship between children's serum magnesium levels and their weight status, with overweight children exhibiting markedly lower average serum magnesium levels compared to normal-weight children, as supported by the presented data. This suggests that low serum magnesium levels may contribute to obesity in children, but further confirmation is required by undertaking large-scale studies. The relationship between metabolic health, magnesium levels, and insulin resistance in children has raised important questions that require additional research. However, it is essential to gain a deeper understanding of the specific mechanisms that underlie this relationship, highlighting the potential importance of magnesium in the context of pediatric obesity.
